# Nutritional Management of Gastrointestinal Diseases and Disorders

**DOI:** 10.3390/nu11123013

**Published:** 2019-12-10

**Authors:** Magdy El-Salhy

**Affiliations:** 1 Section for Gastroenterology, Department of Medicine, Stord Hospital, Box 4000, 5409 Stord, Norway; magdy.el-salhy@helse-fonna.no; Tel.: +47-534-910-00; Fax: +47-534-910-01; 2 Department of Clinical Medicine, University of Bergen, 5021 Bergen, Norway; 3 National Centre for Functional Gastrointestinal Disorders, 5021 Bergen, Norway

Diet not only provides the nutrition necessary for energy and body growth and repair, but also affects and regulates several important functions of the body. The proportions of proteins, carbohydrates and fats in our diet control the type and amount of gastrointestinal hormones released into the bloodstream. These hormones regulate gastrointestinal motility, secretion and absorption, cell proliferation, appetite, and local immune defences [1]. Furthermore, the gastrointestinal hormonal peptides/amines interact and integrate with the enteric, autonomic, and central nervous systems in the so-called gut–brain axis. Food intake also affects the intestinal microbiota, which is believed to play an important role in health and disease. This issue presents the latest research on the use of dietary management to treat gastrointestinal diseases and disorders, and discusses the possible mechanisms underlying its effects.

The role of diet in the pathophysiology of managing irritable bowel syndrome (IBS) dominates this issue. This is not only because a special issue of *Nutrients* was published recently that dealt with diet in inflammatory bowel disease, but also because diet plays an important role in both the pathophysiology and management of IBS. This issue contains one review, two communications, and seven original articles covering several important aspects of this field, and the reported data are novel, interesting, and of high clinical relevance.

## 1. Role of Diet in the Management of IBS

IBS has a lower prevalence and different clinical presentation in Asia compared to the western world, and probably also a different pathophysiology [2,3]. The food dishes typically served in Asia have complex recipes that usually include ingredients rich in several fermentable oligosaccharides, disaccharides, monosaccharides, and polyols (FODMAPs). This situation makes it challenging to evaluate the effect of a low-FODMAP diet effect on Asian patients with IBS. In this issue, the effect of a low-FODMAP diet in Thai IBS patients is compared to the commonly recommended diet, which is a variant of National Institute for Health and Care Excellence-modified diet (NICE-modified diet) [4]. It was found that 60% of the Thai IBS patients responded to a low-FODMAP diet, which is similar to reports that 50–72% of western IBS patients respond to a low-FODMAP diet [5,6,7]. However, the response rate among Asian IBS patients to the NICE-modified diet was 28%, compared to 41–54% in IBS patients in Europe and the USA [5,6,7]. This discrepancy could be explained by the patients randomized to the NICE-modified diet in the present study having received only brief dietary advice (5 min), compared to the structured dietary advice (30 min) provided to the low-FODMAP group, since structured dietary advice itself improves IBS symptoms [8,9,10]. Given the complexity of food dishes in Asia and the tradition of consuming up to six shared different dishes at each meal, recommending a low-FODMAP diet to Asian IBS patients is likely to be difficult.

An open-label study found that 66.3% of IBS patients responded to a diet low in starch and sucrose [11], which is similar to the typical response rate to a low-FODMAP diet. However, both the intervention time (2 weeks) and the observation time were rather short. Intervention studies on IBS patients have a placebo effect of around 40% during the first two weeks following the intervention [12]. Furthermore, the treated patients received dietary guidance, whereas those in the control group did not. Dietary guidance has been reported to improve the symptoms and quality of life in IBS patients [8,9,10], and so it is not clear whether the effect of a low-starch, low-sucrose diet is due to the intervention or the placebo effect added to the effect of the provided dietary information. Further double-blind placebo-controlled studies are needed before any definite conclusion can be drawn.

Wheat is the main source of carbohydrates in the western world, whereas rice and cellophane noodles are the main sources in Asian food. Carbohydrates in rice are completely absorbed in the small bowel and consequently result in the generation of less intestinal gas. In contrast, cellophane noodles are made from mung bean flour, which contains large amounts of oligosaccharides and fructans, but these oligosaccharides are soluble in water and can be eliminated by adequate presoaking during the process of making cellophane noodles. One of the studies in this issue investigates the differences between ingesting wheat (wheat noodles), rice, and cellophane noodles (mung bean noodles) on intestinal gas production and abdominal distension/bloating in non-constipated IBS patients. That study shows that consuming rice and cellophane noodles results in a significantly lower amount of intestinal gas production and less abdominal distension/bloating in non-constipated IBS patients. These results are of considerable clinical importance for diet recommendations to IBS patients and provide scientific support for the recent trend in the western world of IBS patients preferring Asian foods.

Supplements containing fish protein hydrolysates have been reported to beneficially influence several metabolic factors and exert an immune-modulating effect in the gut. One of studies in this issue investigates the effects of consuming cod protein hydrolysate supplement over six weeks on symptom severity, gut integrity markers, and faecal fermentation in IBS patients [13], and found no effect of cod protein hydrolysate compared to the placebo. The required total sample size is 40 patients, with 20 in each arm (α = 0.05; 1 − β = 0.80). However, that study included only 13 patients in the treated arm and 15 patients in the placebo arm, and hence it was clearly underpowered. The effect of cod protein hydrolysate on IBS patients therefore remains to be determined in studies involving larger cohorts of IBS patients.

## 2. Role of Diet in the Pathophysiology of IBS

The low density of enteroendocrine cells in patients with IBS is believed to play a central role in the pathophysiology of the disorder [14]. This low enteroendocrine-cell density appears to be caused by both the low density of intestinal stem cells and the low rate of stem cells differentiation into enteroendocrine cells [15]. In one of the articles in this issue, it is speculated that by-products resulting from bacterial fermentation of the diet interact with gut stem cells causing the low differentiation rate into endocrine cells, leading to the manifestation of gastrointestinal dysmotility, visceral hypersensitivity, and abnormal gastrointestinal secretion [1]. This in turn would give rise to the symptoms characteristic of IBS.

The above-mentioned assumption is supported by the review by Chen et al. published in this issue [16], which reports that glutamine supplements increase the number of intestinal Musashi 1 (a marker for stem cells) cells and chromogranin A (a general marker of enteroendocrine cells). Furthermore, that review reports that glutamine exerts only a mild effect on stem cells, with the main effect of glutamine being on the differentiation activity of the stem cells into both absorptive and secretory lineages [16]. A particularly interesting observation is a dramatic reduction of symptoms in patients with IBS in a recent randomized placebo-controlled trial of dietary supplements containing glutamine [17].

## 3. Role of Probiotics Supplements in Managing IBS

The intestinal bacterial profile in patients with IBS differs from that in healthy subjects, with IBS patients having a lower bacterial diversity (dysbiosis). Patients with IBS who do not respond to dietary management suffer from severe dysbiosis [1]. The article in this issue by Catinean et al. [18] indicates that a mixture of spores from five *Bacillus* spp. improved the symptoms, quality of life, and rectal sensation in patients with IBS to the same degrees as administering a nutraceutical agent or adhering to a low-FODMAP diet [18].

A communication of the literature on the effect of probiotics on IBS symptoms and quality of life in this issue [19] indicates that several questions still need to be answered before recommending the administration of probiotics supplements to patients with IBS in the clinic. For example, we still do not know which bacteria species are the most beneficial for IBS, nor the appropriate dose or for how long they should be administered, or if the same probiotic is suitable for all patients or whether the administered probiotics should be individualized.

Within the limitations of using probiotics supplements to restore the abundance and diversity of the intestinal bacteria, it is conceivable that transplanting the microbiome from a healthy subject with adequate bowel function to IBS patients would constitute an ideal intervention. The application of faecal microbiota transplantation (FMT) in open-label studies involving small cohorts of patients with IBS has produced promising results [20]. However, two recent randomized, double-blind, placebo-controlled studies of FMT produced contradictory results, with one study showing positive results for FMT but the other study finding no effect [21,22]. A recent study [12] showed that FMT is effective in reducing IBS symptoms and fatigue, as well as improving the quality of life of IBS patients. That study showed further that a well-defined donor who is normobiotic and has a special faecal bacterial signature is essential for FMT success. The study also showed that increasing the amount of the transplant and/or repeating FMT increases the response to FMT in patients with IBS.

## 4. Role of Post-Operative Feeding in Gut Surgery

Oesophagectomy is among the most-invasive surgical procedures performed on patients with gastrointestinal carcinoma. The post-operative feeding of patients who have undergone oesophagoectomy is addressed in this issue [23]. That study showed that post-operative per-oral feeding (PO) improves the post-operative nutritional status and overall prognosis compared to enteral nutrition (EN) and parenteral nutrition (PN). Those authors also presented similar data on the effects of colorectal surgery in experimental animals. The results of that study are highly clinically relevant and should be kept in mind when managing the nutrition of patients who are operated on for gastrointestinal carcinoma.

The superiority of PO over EN and PN can be explained by nutrients in the gut lumen stimulating the release of a cascade of neuroendocrine peptides/amines from the gastrointestinal cells. These neuroendocrine peptides/amines regulate the gastrointestinal motility, secretion of enzymes and gall-bladder acids, absorption of nutrients, water and electrolytes, the proliferation of intestinal cells, local immune defences, and appetite. Furthermore, they interact and integrate with the enteric, autonomic, and central nervous systems [1].
